# Barriers to the implementation of programs for the prevention of mother-to-child transmission of HIV: A cross-sectional survey in rural and urban Uganda

**DOI:** 10.1186/1742-6405-2-10

**Published:** 2005-10-28

**Authors:** Francis Bajunirwe, Michael Muzoora

**Affiliations:** 1 Department of Community Health, Mbarara University of Science and Technology, P.O. BOX 1410, Mbarara Uganda; 2 Case Western Reserve University, School of Medicine, Department of Epidemiology and Biostatistics, 10900 Euclid Avenue, Cleveland OH, 44106-4945 USA

## Abstract

**Background:**

Implementation of programs for the prevention of mother-to-child transmission (PMTCT) of HIV faces a variety of barriers and challenges. The assessment of these challenges has generally been conducted in large urban health facilities. As programs expand into rural areas, the potential barriers that may be encountered there also need to be assessed. This study examines potential barriers that might affect the acceptability of interventions for PMTCT in rural and urban settings.

**Results:**

Four hundred and four women at a large urban hospital and three rural clinics that had recently started implementing PMTCT were interviewed. Level of knowledge of MTCT and preference for rapid HIV testing were equally high in both areas, but rural women had a higher tendency to think that they should consult their husbands before testing, with borderline statistical significance (72% vs. 64% p = 0.09). Health facility-based deliveries were significantly lower among mothers in rural areas compared to those in the urban setting. Overall, significant predictors of willingness to test for HIV were post-primary education (OR = 3.1 95% CI 1.2, 7.7) and knowledge about rapid HIV tests (OR = 1.8, 95% CI 1.01, 3.4). The strongest predictor of willingness to accept an HIV test was the woman's perception that her husband would approve of her testing for HIV. Women who thought their husbands would approve were almost six times more likely to report a willingness to be tested compared to those who thought their husbands would not approve (OR = 5.6, 95% CI 2.8, 11.2).

**Conclusion:**

Lessons learned in large urban hospitals can be generalized to rural facilities, but the lower proportion of facility-based deliveries in rural areas needs to be addressed. Same-day results are likely to ensure high uptake of HIV testing services but male spousal involvement should be considered, particularly for rural areas. Universal Primary Education will support the success of PMTCT programs.

## Background

Short course antiretroviral regimens for the prevention of mother-to-child transmission of HIV are cost effective [1], easy to administer, and for these reasons, are being scaled up in many developing countries [2,3]. Despite the low cost for these short course regimens, implementation of programs for the prevention of mother-to-child transmission (PMTCT) of HIV faces many challenges. Some of these challenges include the low uptake of Voluntary Counseling and Testing (VCT) [4-7], failure to return for HIV test results [8] or failure to return for follow up visits before starting antiretroviral therapy [9].

These challenges affect program components differently, and consequently success of each program implementation as a whole has varied quite remarkably. The program components in PMTCT include VCT for HIV during pregnancy, comprehensive antenatal care, infant feeding counseling and administration of short course antiretroviral therapy regimen, intrapartum and postnatal care. For a given program, there may be variation in the success with some program components performing well, while others fail. For example, acceptability of HIV testing may be high but collection of test results and mother-child follow ups are not as successful [10].

Most of the research to assess barriers that may hinder the success of PMTCT program components has been conducted at large urban hospitals [11-14] and it is not clear whether these experiences can be generalized to rural facilities. In many developing countries, the population is predominantly rural and the majority of women seek care at their rural health units. There are significant differences in the socio-demographic structure of populations that live in urban versus rural areas in most of Africa with urban populations being more educated and economically advantaged compared to the rural population. A recent study from the Ivory Coast has shown that socio-demographic factors may be associated with participation by HIV positive women in an intervention for PMTCT [15]. The objective of this survey was to assess knowledge of Mother-to-Child Transmission (MTCT) of HIV and to describe the potential barriers that might affect acceptability of interventions for PMTCT, particularly rapid testing for HIV and short course antiretroviral therapy among mothers in rural and urban settings.

## Methods

This is a cross-sectional study conducted over a period of four months between September and December 2003. Face to face interviews were administered to mothers attending antenatal clinics in rural and urban parts of Mbarara, a district in southwestern Uganda. The PMTCT program in Mbarara district started in August 2002 at Mbarara University Hospital, the regional referral hospital. In February 2003, a scale-up program to the peripheral rural health units started, with support from Elizabeth Glaser Pediatric AIDS Foundation. When this survey was conducted, the program had been in operation for one year at the urban hospital and six months at the rural health units.

Mothers were enrolled consecutively for face to face interviews from Mbarara University Hospital antenatal clinic, an urban setting, and also from three rural health units at county level in Ibanda, Bwizibwera, and Kazo, that were each implementing PMTCT.

Mothers received information about the study during their visit to the antenatal clinic and were requested to participate. Informed consent was obtained by a trained counselor, who carried out the interview as well.

### Sample size and analysis

Survey items were socio-demographic characteristics, knowledge on mother-to-child transmission of HIV, attitudes towards voluntary counseling and testing (VCT) and mothers' willingness or intention to accept rapid testing for HIV if it were offered. Mothers were enrolled consecutively until the required sample size was achieved. However, as part of the survey, the mothers were not followed to determine those who eventually accepted HIV testing. Sample size calculation was based on estimates for the level of acceptance of rapid HIV tests in rural and urban areas. We hypothesized that the intention to accept rapid HIV tests would be higher in urban health units compared to rural units. At the time the survey was designed, VCT acceptance rates using rapid tests were 60% at antenatal clinics in urban settings [16] and we projected the acceptance rates to be 45% in the rural setting. Using a two sided alpha level of 5%, a sample size of 165 per group would provide a power of 80% to detect a difference of 15% in the proportion of those willing to accept HIV rapid tests.

Data was entered into EPIDATA software, and analysis was performed using SPSS (Version 13 for Windows). Chi square testing was used to test for differences in demographic characteristics between rural and urban mothers. Chi square tests were also conducted to test for differences in perceptions and attitudinal factors between the two groups. Unconditional logistic regression was performed to determine the factors that predicted the intention to accept rapid HIV testing.

## Results

### Demographics

Four hundred and four pregnant women attending four antenatal clinics where PMTCT services were offered at the time were interviewed. The antenatal clinic in Mbarara University Hospital is located in an urban setting, while the other three clinics are rural. Of the 404 respondents, 212 (52%) were urban, while 192 (48%) were rural. The majority of respondents had received some schooling (369 or 91%), while only 35 (9%) had never been to school. In addition, literacy among the respondents was high, with 348 or 88% of the women able to read and 332 or 86% of them able to write. Most of the women were married and living with their spouse (348 or 88%), while 44 (11%) were single mothers and the remainder were separated, divorced or widowed. The number of women pregnant for the first time (prime gravida) was the same in the rural and urban areas. Radio ownership was high in both rural and urban areas but higher in urban areas. Table 1 shows the demographic characteristics stratified by location of the clinic.

**Table 1:** Demographic characteristics of 404 rural and urban women interviewed at four clinics in Mbarara, Uganda

	Rural n = 212 n(%)	Urban n = 192 n(%)	*p *value
Mean age	24.2	24.6	0.43^+^
Mean number of pregnancies	3.3	3.0	0.19^+^
Ever been to school	174 (91)	194 (92)	0.72
Have post primary education	42 (22)	62 (30)	0.09
Can read	166 (88)	182 (88)	0.98
Can write	152 (81)	180 (88)	0.42
Own a bicycle	106 (68)	117 (60)	0.14
Own a radio	166 (88)	199 (96)	0.005
Listen to radio	166 (88)	198 (95)	0.018
Prime gravida	53 (28)	49 (24)	0.32

^+ ^Obtained using t-test for independent means. The other *p *values are from chi square tests for independence

### Knowledge about MTCT and prevention

Overall knowledge regarding MTCT was high, and 325 (80%) knew that a mother with HIV can pass the virus to her child. However, 47 (12%) mothers interviewed did not think that it was possible for the virus to be passed to the unborn baby, and the remaining 8% did not know whether the virus can be passed from mother to child. The survey shows that 159 (83%) mothers in the rural area knew that MTCT can occur compared to 166 (81%) mothers in the urban area (Chi square *p *= 0.77, df = 2). These figures demonstrate that the level of knowledge did not differ significantly between the mothers in the rural setting compared to those in the urban areas.

The mothers were also asked whether HIV could be transmitted through breast milk and overall, 268 (77%) knew that breast transmission of HIV was possible. Thirty eight (11%) thought that breast transmission of HIV was not possible and 41 (12%) did not know whether breast milk could cause HIV transmission or not. Fifty seven mothers did not answer this question and therefore were not evaluated for this response.

There were no significant differences in the level of knowledge between the rural and urban mothers regarding breast milk as a possible route of transmission (Chi square *p *= 0.65).

Two hundred and eighty six (80%) mothers who responded knew that transmission of HIV from mother-to-child could be prevented. There was no difference in terms of knowledge that mother-to-child HIV transmission can be prevented (Chi square *p *= 0.78) between the rural and urban mothers.

### Attitudes towards HIV testing and, acceptability of rapid HIV testing

Overall, only 89 (22%) women interviewed in the survey had ever been tested for HIV. The willingness to take an HIV test was high with a total of 337 women (87%) responding that they would accept an HIV test if it was offered to them. A significant proportion of mothers (n = 159 or 40%) did not know about the existence of rapid tests for HIV. Nevertheless the majority of mothers (n = 353 or 88%) preferred having same day results from an HIV test, while the rest preferred to receive the results at a later date. In addition, the majority of women (n = 389 or 97%) said they would advise someone to take an HIV test and thought it was beneficial.

The data was analyzed using contingency tables to explore differences between the urban and rural women in regards to knowledge, attitudes and acceptability of HIV testing. The results show that there was no significant difference in the proportion of mothers who knew about rapid tests for HIV, no difference among preference for same day results and also no difference in terms of the proportion of mothers that had ever been tested for HIV, in either the rural or urban areas. Results are shown in Table 2. In addition, mothers in both regions were equally likely to accept medications for the reduction of MTCT if offered, and acceptance rates for these medications were high.

**Table 2:** Knowledge, attitudes and acceptance for rapid HIV testing among rural and urban mothers in Mbarara, Uganda

Variable	Rural n = 212 n(%)	Urban n = 192 n(%)	p* value
Knew about rapid tests for HIV	115 (61)	124 (59)	0.58
Would accept HIV test if offered	159 (89)	178 (86)	0.40
Ever been tested for HIV	41 (24)	48 (23)	0.88
Think it is important to test for HIV	163 (96)	195 (98)	0.55
Would prefer same day results	169 (89)	184 (88)	0.57
Would advise someone to take an HIV test	184 (98)	205 (98)	0.88
Heard a radio program on MTCT	152 (81)	175 (84)	0.45
Had health talk from health worker on MTCT	91 (48)	90 (43)	0.35
Husband aware she came to antenatal today	166 (91)	173 (87)	0.18
Believe should consult husband before HIV test	132 (72)	132 (64)	0.09
Husband may not approve of testing	31 (18)	41 (21)	0.53
Husband would accept HIV test for himself	122 (71)	138 (73)	0.60
Would accept medication for PMTCT	171 (98)	181 (99)	0.50
Think that pregnancy should be terminated if mother is HIV infected	37 (20)	37 (17)	0.60

* All *p *values are obtained from chi square tests for independence

### Role of male partner

For the women living with their husbands, the majority of women (339 or 89%) informed them that they had come to the antenatal clinic that day and also 68% (264) of the women thought that they should consult their husbands before having an HIV test. In addition 81% (299) of the women thought that their husbands would approve of their being tested and the remaining (n = 72 or 19%) feared that their husbands would not approve of their being tested. Also, the majority of women (n = 260 or 72%) thought that their husbands would accept the HIV test for themselves.

The analysis shows that the rural and urban mothers were equally likely to inform their husbands that they had come to the antenatal clinic that day (Chi square *p *= 0.18). There was no significant difference in the proportion of mothers who thought they should consult their husbands before they were tested for HIV in the rural and urban areas, though there is a tendency for the rural women to think they should consult their husbands before testing as shown in table 2 (72%vs. 64% chi square p = 0.09).

### Predictors of willingness to accept HIV testing

In a univariate logistic regression analysis, different variables were examined to determine the factors that predict intention or willingness to accept HIV testing and the results are shown in Table 3. In the analysis, mothers who had an education beyond seven years of primary school were almost three times more likely to report a willingness to be tested compared to those who had not finished primary school education or had not been educated at all (Odds Ratio OR= 2.8, 95% Confidence Interval, CI 1.2, 6.9.). Also, mothers who are able to read were two times more likely to report a willingness to be tested compared to those who cannot read (OR= 2.2, 95% CI 1.02, 4.9). The ability to write was an even stronger predictor with mothers who could write three times more likely to report willingness to accept HIV testing than those who could not write (OR = 2.9, 95% CI 1.4, 6.0). The knowledge as to whether mother-to-child transmission of HIV can occur and the number of previous pregnancies were not significant predictors of the mothers' intention to accept HIV testing. However knowledge that rapid HIV tests exist and that someone can be tested and receive results the same day was a significant predictor (OR = 1.9, 95% CI 1.01, 3.4).

**Table 3:** Univariate Logistic regression analysis to demonstrate the factors associated with willingness to accept HIV testing among antenatal mothers in Mbarara, Uganda

Variable	Odds ratios (95% CI)
Age	
25 years or younger	1.0
Over 25 years	0.88 (0.47, 1.63)

Site	
Urban	1.0
Rural	1.3 (0.71, 2.4)

Educational level:	
Primary or less	1.0
Post primary	2.8 ^++ ^(1.2, 6.9)
Can read	
No	1.0
Yes	2.2^++ ^(1.02, 4.9)
Can write	
No	1.0
Yes	2.9^++ ^(1.4, 6.0)
Knows MTCT can occur	
No	1.0
Yes	1.02 (0.5, 2.2)
Knows about rapid HIV testing	
No	1.0
Yes	1.9^++ ^(1.01, 3.4)
Listens to Radio	
No	1.0
Yes	1.89 (0.73, 4.9)
Thinks woman should consult husband before HIV test	
No	1.0
Yes	0.6 (0.3, 1.2)
Husband would approve of testing for HIV	
No	1.0
Yes	5.6^++ ^(2.8, 11.2)
Number of pregnancies	
First	1.0
Two or more	1.01 (0.51, 2.0)

++ Significant at 0.05 level

Women who thought they should consult their husbands before they were tested for HIV, were 40% less likely to express willingness to accept the test compared to those who thought they do not need to consult their husbands, but this difference was not significant (OR = 0.6, 95% CI 0.3, 1.2).

The strongest factor predicting the willingness or intent to accept an HIV test was the woman's perception that the husband would approve of her being tested. The women who thought their husbands would approve were almost six times more likely to report a willingness to be tested compared to those who thought their husbands would not approve (OR = 5.6, 95% CI 2.8, 11.2).

### Logistic regression analysis

Age has been shown to be a significant factor in the determination of whether mothers will accept HIV testing because of higher risk perception among older women [17]. In this survey, with age analyzed as a dichotomous variable using 25 years as the cut off, age was not associated with reported willingness to accept HIV testing (OR= 0.87 and 95% CI 0.47, 1.62),. Therefore, age was not considered to be a confounder in this study. The proportion of mothers who listen to or own a radio was unequally distributed among the rural and urban areas. Women who listened to the radio were more likely to express willingness to be tested for HIV compared to those who did not, but the association was not significant OR = 1.89 (95% CI 0.73, 4.9). For this reason radio ownership or radio listening were not considered as confounding variables in the assessing the relationship between rural or urban location of women and willingness to accept HIV testing. In the assessment of factors associated with the willingness to accept HIV testing, no confounding factors were identified and therefore only a univariate analysis was performed. Results are shown in Table 3.

### Other barriers

For optimal antiretroviral prophylaxis, it is preferred that the mother delivers her baby in the hospital so the infant can receive his/her prophylaxis. For this reason the mothers who had been pregnant before were asked where they delivered their last pregnancy. One hundred and twelve (39%) reported they had delivered at home, 19 (7%) had been delivered by the traditional birth attendant and 148 (52%) had delivered in a health facility. In a stratified analysis by location, women in the urban area were more likely to have delivered their last child at a health unit compared to those in the rural areas (97 or 62% vs. 51 or 42%). Rural women were more likely to have home deliveries or delivery by the traditional birth attendant (p < 0.0001).

## Discussion

As many countries in the developing world roll out programs for the prevention of mother to child transmission of HIV, there is need to consider the potential barriers that these programs may face. In addressing these barriers, it is crucial that any differences between rural and urban areas are addressed since the significant proportion of people in developing countries live in the rural areas. This study has shown that there are no major differences in terms of the potential barriers that might hinder the success of implementation of PMTCT programs in rural areas as compared to urban areas. This indicates that experiences learned from programs in the urban areas will apply to rural PMTCT programs.

One major challenge identified is that a significant proportion of mothers deliver outside the health facility, and this occurs more frequently in the rural areas compared to the urban areas. Health facility-based delivery is helpful to ensure compliance to infant antiretroviral dosing but also to ensure the practice of modified obstetric practices that have been shown to reduce MTCT [18].

Though rural and urban populations are perceived as differing in knowledge, readiness and ability to follow advice [19], this study suggests the contrary in regards to MTCT. The level of knowledge was high and the readiness to accept HIV testing was equally high in both rural and urban areas. This high level of knowledge may be attributed to various programs being broadcast on the radio in this district, reaching even the distant rural areas, where some of the study participants reside. Radio ownership was high in both rural and urban areas and the proportion of mothers listening to the radio was also high. PMTCT programs should utilize this medium of communication in areas where it is available.

Most of the mothers interviewed preferred same day HIV test results however some mothers preferred to receive results later. It has been shown that same day results can be provided in counseling without compromising the quality of counseling and testing [20]. It is possible that the mothers who prefer to receive results later may be the ones who decline to test for HIV when the test offered is rapid, or may undergo the test but not receive their results. However more studies are required to explore this hypothesis. In the meantime, PMTCT programs should identify the mothers who are likely to refuse testing or would prefer to receive their results at a later date and design a customized schedule to accommodate them since they may be at a higher risk [21]. Conversely, some studies have indicated that those who refuse testing may actually be at lower risk for HIV [22,23].

Many mothers understand that there is a benefit in taking an HIV test as indicated by the large number who said that they would advise someone else to take an HIV test. This proportion is larger than those who said that they would accept an HIV test themselves if it were offered (98% vs. 89% respectively). There is a gap between knowledge about the benefit and acceptance to have the HIV test done. Though there is an almost universal recommendation from the mothers to take the test themselves, not all of them will choose to have the test for themselves.

Whereas some studies have shown that a lower education level is associated with higher likelihood to request for HIV testing [24], this study showed the opposite, with those having at least a post-primary education more likely to choose to test compared to those with lower education. These study findings are supported by a study among Hispanic farm workers in South Florida [25] in which participants with at least twelve years of education were four times more likely to test compared to those without the same education. In a Vietnamese study, low education was associated with not returning for results [26]. The Universal Primary Education campaigns currently underway in some developing countries like Uganda [27] may facilitate implementation of health programs such as PMTCT.

This study demonstrates that male partners' attitudes are important in a woman's reported willingness to accept HIV testing. In some circumstances women have tested for HIV without their husbands consent and have suffered domestic violence [28]. In this survey, the perception that the husband would approve of a mother's decision to test for HIV was the strongest predictor of whether the mother had the intention of testing or not. This finding highlights the importance of the male partner in the success of uptake of HIV testing within PMTCT programs. This study demonstrates that there is a tendency for more rural women to seek their husbands' approval prior to testing compared to their urban counterparts. This may be an inhibitory factor to the willingness to accept VCT. This study reinforces the recommendations made by a study in Tanzania [29] that emphasized the role of the male partner in PMTCT.

One limitation of this survey is that mothers were questioned regarding their willingness to accept HIV testing, but were not followed to determine those who eventually accepted the HIV test. This would have enabled us to establish the relationship between willingness to take the test if it were offered and actually taking it. Actual acceptance of HIV testing would be more informative than answers to the question about willingness to accept testing. In addition, the rural sites chosen for the survey were those that were implementing PMTCT in an ongoing scale-up program at the time the survey was conducted. Since they were not randomly selected, it is possible that these clinics may not be representative of other rural areas in the district. Additionally, the survey was based at the health facility and therefore only mothers seeking antenatal care at a health unit were eligible for the study. Whereas this may be a limitation, it may not be a strong factor in this study because over 80% of women in Uganda seek at least one antenatal visit at a health facility during their pregnancy [30].

## Conclusion

Lessons learned in the implementation of PMTCT programs in urban areas can easily be generalized to rural areas since there are no major differences in terms of attitudes towards acceptance of interventions for PMTCT. Willingness to accept the PMTCT program is high in both rural and urban health units but the lower proportion of births occurring at the health facility, particularly in rural areas, may be a barrier to ensuring neonatal antiretroviral dosing. Same day results for HIV is likely to result in increased uptake of VCT but male partner involvement, particularly in the rural areas, should be considered for complete success of PMTCT programs.

## Competing interests

The author(s) declare that they have no competing interests.

## Authors' contributions

Concept protocol: FB, MM

Data collection: MM

Data analysis: FB

Manuscript draft: FB, MM
